# Select Biomarkers on the Day of Anterior Cruciate Ligament Reconstruction Predict Poor Patient-Reported Outcomes at 2-Year Follow-Up: A Pilot Study

**DOI:** 10.1155/2018/9387809

**Published:** 2018-07-19

**Authors:** Christian Lattermann, Caitlin E.-W. Conley, Darren L. Johnson, Emily K. Reinke, Laura J. Huston, Janet L. Huebner, Ching-Heng Chou, Virginia B. Kraus, Kurt P. Spindler, Cale A. Jacobs

**Affiliations:** ^1^Brigham and Women's Hospital, Harvard Medical School, Chestnut Hill, MA, USA; ^2^University of Kentucky, Lexington, KY, USA; ^3^Vanderbilt University, Nashville, TN, USA; ^4^Duke University, Durham, NC, USA; ^5^Cleveland Clinic Sports Health Center, Garfield Heights, OH, USA

## Abstract

**Background:**

The majority of patients develop posttraumatic osteoarthritis within 15 years of anterior cruciate ligament (ACL) injury. Inflammatory and chondrodegenerative biomarkers have been associated with both pain and the progression of osteoarthritis; however, it remains unclear if preoperative biomarkers differ for patients with inferior postoperative outcomes.

**Hypothesis/Purpose:**

The purpose of this pilot study was to compare biomarkers collected on the day of ACL reconstruction between patients with “good” or “poor” 2-year postoperative outcomes. We hypothesized that inflammatory cytokines and chondrodegenerative biomarker concentrations would be significantly greater in patients with poorer outcomes.

**Study Design:**

Prospective cohort design.

**Methods:**

22 patients (9 females, 13 males; age = 19.5 ± 4.1 years; BMI = 24.1 ± 3.6 kg/m^2^) previously enrolled in a randomized trial evaluating early anti-inflammatory treatment after ACL injury. Biomarkers of chondrodegeneration and inflammation were assessed from synovial fluid (sf) samples collected on the day of ACL reconstruction. Participants completed Knee Injury and Osteoarthritis Outcome Score (KOOS) and International Knee Documentation Committee (IKDC) questionnaires two years following surgery. Patients were then categorized based on whether their KOOS Quality of Life (QOL) score surpassed the Patient Acceptable Symptom State (PASS) threshold of 62.5 points or the IKDC PASS threshold of 75.9 points.

**Results:**

Patients that failed to reach the QOL PASS threshold after surgery (n = 6, 27%) had significantly greater sf interleukin-1 alpha (IL-1*α*; p = 0.004), IL-1 receptor antagonist (IL-1ra; p = 0.03), and matrix metalloproteinase-9 (MMP-9; p = 0.01) concentrations on the day of surgery. Patients that failed to reach the IKDC PASS threshold (n = 9, 41%) had significantly greater sf IL-1*α* (p = 0.02).

**Conclusion:**

These pilot data suggest that initial biochemical changes after injury may be an indicator of poor outcomes that are not mitigated by surgical stabilization alone. Biological adjuvant treatment in addition to ACL reconstruction may be beneficial; however, these data should be used for hypothesis generation and more definitive randomized clinical trials are necessary.

## 1. Introduction

Whether isolated or in concert with concomitant meniscal or articular cartilage injury, anterior cruciate ligament (ACL) rupture initiates a cascade of cytokine and catabolic enzyme activity [1–4]. Regardless of surgical or conservative management, the majority of patients develop posttraumatic osteoarthritis (PTOA) within 15 years of ACL injury [5–7]. In addition to magnetic resonance imaging (MRI) and/or radiographic evidence of cartilage degeneration [8–11], the onset of PTOA is associated with increased concentrations of chondrodegenerative and inflammatory biomarkers [12, 13]. Increased biomarkers of cartilage turnover have not only been reported as a late consequence of ACL injury, but also been reported to be significantly elevated immediately following ACL injury [2–4, 14]. This may indicate chronically increased cartilage turnover and a progressively destructive process that may have long-term clinical consequences. However, while inflammatory and chondrodegenerative biomarkers are elevated in the period between ACL injury and reconstruction [3, 4], there is a substantial knowledge gap with regard to the role of preoperative biomarker concentrations and their association with either successful or suboptimal clinical outcomes.

One method to objectively quantify a successful outcome is to determine whether a patient has achieved a Patient Acceptable Symptom State (PASS) following surgery [15–18]. The PASS represents a threshold for a given postoperative outcome score above which the patient is defined as having a satisfactory outcome. Patients that fail to achieve the PASS threshold are then considered to have a suboptimal postoperative outcome. The purpose of this pilot study was to compare biomarkers assessed from samples collected preoperatively on the day of ACL reconstruction between patients with 2-year postoperative patient-reported outcome scores above and below previously established PASS thresholds [18]. We hypothesized that synovial fluid and urine concentrations of inflammatory cytokines and chondrodegenerative biomarkers would be significantly greater in patients with poorer outcomes.

## 2. Methods

Participants included 22 patients (9 females, 13 males; age=19.5 ± 4.1 years (mean ± standard deviation); BMI=24.1 ± 3.6 kg/m^2^, Table 1) that had previously consented to participate at one site of an IRB-approved, multicenter prospective randomized clinical trial evaluating early anti-inflammatory treatment after ACL injury (clinicaltrials.gov ID: NCT01692756) [3]. At the time of enrollment, patients with an isolated ACL injury between the ages of 14 and 32 with closed growth plates were included. Other inclusion criteria included a normal contralateral knee, no history of previous traumatic ipsilateral knee injury, and an ACL injury that occurred during sports activity with no clinical evidence of posterior cruciate ligament injury and with no more than grade 1 medial or lateral collateral ligament injury. Patients were excluded if the injury occurred more than eight days prior to enrollment or they had a history of previous ipsilateral knee surgery, a known allergy to triamcinolone acetonide, intra-articular cortisone injection into either knee within three months of injury, or a history of any inflammatory disease or immunocompromise [3].

In the initial trial, biomarkers were assessed at three time points prior to surgery to determine if early anti-inflammatory treatment could alter the expected progressive increases in inflammatory and chondrodegenerative biomarker concentrations [3]. The three time points from the initial study were (1) mean 4 days after injury, (2) mean 11 days after injury, and (3) the day of ACL reconstruction (mean 37 days after injury). For the current study, we used only biomarker data from samples acquired on the day of ACL reconstruction to assess if inflammatory and chondrodegenerative biomarker concentrations differed between those with good versus poor postoperative outcomes. In original study, patient-reported outcomes on the day of surgery did not differ between patients treated with preoperative intra-articular corticosteroid and the placebo group. In addition, the surgical intervention and postoperative rehabilitation and medications did not differ between groups and no between-group differences were noted in either postoperative Knee Injury and Osteoarthritis Outcome Score (KOOS) Quality of Life scores (QOL; corticosteroid = 72.7 ± 16.1 versus placebo = 67.8 ± 31.1, p = 0.82) or International Knee Documentation Committee scores (IKDC; corticosteroid = 74.8 ± 12.9 versus placebo = 71.2 ± 12.3, p = 0.46). As such, we therefore pooled data for all patients for the current analysis.

Knee aspiration was performed aseptically at the time of surgery via a superolateral suprapatellar approach, and intra-articular placement was confirmed using the “squish” test [19]. Patients were fasting as both urine and synovial fluid (sf) samples were collected on the morning of surgery. Synovial fluid and urine samples were spun at 3500 RPM for 10 minutes and the supernatant aliquoted according to an allotment protocol. Once all samples were collected they were shipped to the Duke University biomarker laboratory (VBK/JLH) for analysis [3].

A number of chondrodegenerative biomarkers and inflammatory cytokines were assessed using previously described methods (Table 2) [3, 4]. All biomarker concentrations were assessed from synovial fluid samples with the exception of CTX-II which was assessed in both the synovial fluid (CTX-II) and urine (uCTX-II). Urinary CTX-II was normalized to creatinine concentration (ng/mmol) [13]. Biomarker variability, linear range of standard, and need for repeat were carefully assessed. Several commercially-available ELISA kits provided controls, which were used with every run to confirm that values were within the acceptable range according to the manufacturer and to assess interassay (plate to plate) variability. In addition, synovial fluid from a human subject with knee OA that had previously been aliquoted and frozen at −80°C was used as the control for all assays. A fresh aliquot of this control synovial fluid was thawed and used on every plate to calculate intra- and interassay variance of the assay each day that analyses were performed. Due to limited sf sample volume available, samples were run in singlicate; however, all standards and controls were run in duplicate and used to determine the precision of the assay and to establish an acceptable control range for the assay. The mean of the control sample for all assays plus or minus two standard deviations was defined as the acceptable control range. All control samples were within the acceptable control range for all plates and assays. Mean intra-assay coefficients of variation for each assay are reported in Table 2. Mean interassay coefficients of variation were all <15% with the exception of IL-1*α* and IL-1*β*. The manufacturer reported that interassay CVs for these assays are 6.6% and 6.4%, respectively; however due to the human control SF sample having values at or below the level of detection, this calculation was not possible. For values of IL-1*α* and IL-1*β* that were below the lower limit of detection, 1/2 LLOD (lowest level of detection) was reported for statistical purposes.

Patients were contacted two years after surgery to complete standardized patient-reported outcomes, including IKDC and KOOS Scores [18, 21–25]. The volume of preoperative bone bruises was calculated from each patient's preoperative magnetic resonance imaging (MRI) scan using previously described methods [26]. MRIs were considered adequate for inclusion if they included T2 or PD sequences in the coronal, axial, and sagittal plane as well as slice thicknesses < 5 mm. The bone bruise volumes in the medial tibial plateau, medial femoral condyle, lateral tibial plateau, and lateral femoral condyle were measured from the T2 or PD weighted coronal images in a modification of Roemer and Bohndorf's technique [26, 27]. Bone bruises volumes from each of the four bony regions were then summed and expressed as the total bone bruise volume (mm^3^) [26].

### 2.1. Statistical Analysis

Mann–Whitney U tests were performed to determine if inflammatory cytokines and chondrodegenerative biomarkers assessed from samples collected on the day of surgery (Table 2) differed from those with either a good or poor outcome based on postoperative IKDC and KOOS QOL scores. Due to the high number of IL-1*β* samples that were below the lower limits of detection (11/22, 50%), for this cytokine we also used Fisher Exact tests to compare the prevalence of concentrations below the lower limits of detection (LLOD) between groups. Patients were categorized as having a good or poor outcome based on whether they reported IKDC or KOOS QOL scores above or below previously established Patient Acceptable Symptom State (PASS) thresholds following ACL reconstruction of 62.5 points for KOOS QOL and 75.9 points for the IKDC [18]. As the KOOS QOL and IKDC may be assessing different aspects of the patient's outcome, separate analyses were run based on the KOOS QOL PASS definition and IKDC PASS definition. Cohen's d effect sizes calculations were also performed in order to identify potentially clinically-meaningful findings within these pilot data, with d > 0.80 considered a large effect size [28]. Analyses were performed using SPSS Statistics 24 (IBM, Armonk, NY) and Excel 2016 (Microsoft, Redmond, WA) with p < 0.05 considered statistically significant. All data and results are stored at the Department of Orthopaedic Surgery and Sports Medicine at the University of Kentucky.

## 3. Results

At a mean follow-up of 2.4 years, 6/22 patients (27%) had postoperative KOOS QOL scores below the PASS threshold of 62.5 points (Table 3). Patients that failed to reach the QOL PASS threshold had significantly greater sf IL-1*α* (p=0.004), sf IL-1ra (p=0.02), and sf MMP-9 (p=0.01). While not statistically significant, markers of type I and type II collagen breakdown (sf NTX-I [p=0.055] and uCTX-II [p=0.08]) tended to be greater for those that failed to reach the QOL PASS threshold. Graft type, the prevalence of meniscus injury, and bone bruise volumes did not differ between QOL PASS groups (Table 3).

Nine of 22 patients (41%) failed to reach the IKDC PASS threshold of 75.9 (Table 4). Patients that failed to reach the IKDC PASS threshold had significantly greater sf IL-1*α* (p=0.02). While not statistically significant, sf IL-1ra (p=0.057), sf MMP-1 (p=0.10), and sf MMP-9 (p=0.10) tended to be greater for those that failed to reach the IKDC PASS threshold. No other biomarker differences were noted between those that did or did not surpass the IKDC or QOL PASS thresholds. Graft type, the prevalence of meniscus injury, and bone bruise volumes did not differ between IKDC PASS groups (Table 4).

## 4. Discussion

The purpose of this pilot study was to compare biomarkers assessed from samples collected on the day of ACL reconstruction between patients with postoperative patient-reported outcome scores above and below previously established PASS thresholds [18]. We hypothesized that synovial fluid and urinary concentrations of inflammatory cytokines and chondrodegenerative biomarkers would be significantly greater in patients with poorer outcomes, and our hypotheses were, in part, supported by the current results.

The current pilot results support the previously described cascade of increased IL-1*α* stimulating the production of matrix metalloproteinases thereby reducing proteoglycan content and altering cartilage mechanical properties. Previous studies have demonstrated that ACL injury triggers a biochemical cascade that worsens over the first 4-6 weeks after injury [2–4]. Inflammation is initiated early by the injury-related hemarthrosis and subsequent intra-articular pathogenic processes at the time of injury, including the downregulation of proteoglycan synthesis and upregulation of matrix metalloproteinases [29–31]. It is widely accepted that IL-1 and IL-1ra are critical in the regulation of the pathological processes involved in joint tissue breakdown [32, 33]. Synovial fluid IL-1 levels are elevated in patients with ACL rupture and correlate with severity of chondral damage [34]. Synovial fluid levels of the chondroprotective IL-1ra cytokine are reported to decrease significantly after ACL injury, resulting in relatively unopposed activity of IL-1 [1]. Additionally, levels of IL-1ra decrease as the severity of chondral damage is increased [34]. The superficial cartilage layers have been shown to be more susceptible to IL-1 induced damage than deeper layers* in vitro *[35]. Porcine cartilage explants have been demonstrated to be more sensitive to the chondrodegenerative effects of IL-1*α* than IL-1*β* [36].

The current pilot results suggest that initial biochemical changes after injury may be prognostic of long-term consequences of injury of the knee that are not mitigated by surgical stabilization alone. Inflammatory cytokine and degradative enzyme concentrations on the day of ACL reconstruction have been previously shown to correlate with cartilage changes on MRI during the first three years after surgery [37], and the current results further demonstrate a connection between cytokine and degradative enzyme concentrations and the potential progression of posttraumatic OA. In the current study, both IL-1*α* and MMP-9 concentrations on the day of surgery were significantly higher for patients that failed to postoperatively achieve the KOOS QOL-based PASS compared to those with better clinical outcomes. In addition to increased IL-1a and MMP-9 concentrations, biomarkers of bone and cartilage turnover (sf NTX-I, d = 0.97 and uCTX-II, d = 0.99) were also increased for patients that failed to achieve KOOS QOL-based PASS. This overall state of upregulated catabolism and inflammation may lead to recurrent effusions and/or persistent synovitis which also appears to potentially have long-term implications for knee health.

In addition to playing a contributing role in the progression of structural posttraumatic OA changes [37], increased inflammatory cytokine and degradative enzyme concentrations on the day of ACL reconstruction also appear to affect postoperative patient-reported outcomes. In OA knees, nerve growth factor expression in the synovium has been shown to modulate pain by increasing local nociceptor sensitization [38, 39]. Nerve growth factor expression is increased in the synovium of OA knees [40], and by regulating nerve growth factor expression, proinflammatory cytokine IL-1 and other degradative enzymes may play an integral role in modulating knee pain [40]. While attention is given the potential role of the innate immune response on cartilage degradation and the progression of OA [41], it should be noted that, after adjusting for age, peripheral cytokine concentrations are similar between patients with knee OA and fibromyalgia [42]. Hypersensitivity and nociceptor sensitization secondary to increased proinflammatory cytokine concentrations may then explain the increased IL-1*α* and MMP-9 concentrations for those that failed to achieve a Patient Acceptable Symptom State in the current study.

Early anticatabolic and/or anti-inflammatory interventions after ACL injury or reconstruction may need to be further investigated as adjunctive treatment strategies to mitigate both the structural changes and symptoms of posttraumatic OA. CTX-II has been reported to be predictive of OA progression based on both radiographic and arthroscopic evidence [43–45]. Particularly relevant to the current results suggesting a link between CTX-II concentrations and patient-reported outcomes, Ishijima et al. reported that urinary CTX-II was associated with concurrent OA-related pain [46]. The association between CTX-II, pain, and inferior outcomes may be tied to the combination of both changes to the articular cartilage and to bone metabolism. Garnero et al. first reported that urinary CTX-II concentrations in early OA patients were predictive of bone marrow lesion progression [47]. More recently, in a cluster analysis of a wide spectrum of OA-related biomarkers, van Spil reported that CTX-II tended to cluster with other biomarkers associated with bone metabolism [48]. Bone marrow lesions are common following ACL injury [26, 49], and many resolve over time [50]. However, subjective symptoms of early OA are more common six years after ACL reconstruction for those noted to have local articular cartilage damage combined with bone marrow lesions at the time of surgery [26]. The size and progression of bone marrow lesions have been previously linked to OA-related pain as the subchondral bone is rich with nociceptors whereas the articular cartilage is not [51–53]. Taken together with our data, links between CTX-II, NTX-I, IL-1*α*, pain, and both cartilage and bone metabolism may all contribute to KOOS QOL and IKDC outcomes at 2 years.

This study had several limitations. First and foremost, the results of this preliminary work are based on a small sample size and additional studies are necessary to confirm these results. Second, some patients had received corticosteroid injections prior to surgery which could have influenced biomarker concentrations on the day of surgery. As mentioned previously, KOOS QOL and IKDC scores did not differ between patients treated with preoperative intra-articular corticosteroid and the placebo group, and the surgical intervention and postoperative rehabilitation and medications did not differ between groups and no between-group differences were noted in either postoperative KOOS QOL or IKDC scores. Third, the prevalence of patients with postoperative KOOS QOL and IKDC scores below the PASS threshold was higher in the current study than those originally reported by Muller et al. [18]. This may be due, in part, to the differences in patient populations between the two studies. In the current study, all patients were injured during athletic participation with an average age at the time of surgery of 19.5 years compared to 26.1 in the study by Muller et al. [18]. The lingering effects of athletic injuries have been reported to negatively impact quality of life years after competition has ended, and both physical and mental aspects of function are significantly lower in former collegiate athletes compared to college attendees who did not participate in sport [54]. It remains unclear if the increased prevalence of patients with inferior patient-reported outcomes in the current study was perhaps due to the strict inclusion criteria of athletic ACL injuries. Fourth, while inferior patient-reported outcome scores have been associated with early OA changes, postoperative imaging or biomarker evidence of OA will be necessary to establish the potential connection between biomarkers on the day of surgery, KOOS QOL, IKDC scores, and evidence of structural features of knee OA. Finally, future work may require more sensitive methods to quantify synovial fluid IL-1*β* concentrations as 50% of the samples in the current study had values below the lower limits of detection.

In conclusion, the results of this preliminary investigation demonstrated that higher inflammatory cytokine activity (IL-1*α*) and a trend towards greater bone and cartilage collagen degradation (NTX-I and uCTX-II) at the time of surgery were associated with failure to achieve an acceptable symptom state two years after ACL reconstruction. These data suggest that initial biochemical changes after injury may be prognostic of long-term consequences of ACL injury that are not mitigated by surgical stabilization alone; however, due to the small sample size these data should be used for hypothesis generation and more definitive randomized clinical trials are necessary.

## Figures and Tables

**Table 1:** Concomitant injuries and surgical treatments (n (%)) performed at the time of ACL reconstruction.

	**Not injured**	**Injured, not treated**	**Debrided**	**Repaired**
Meniscus				
Medial	8 (36%)	0 (0%)	1 (5%)	13 (59%)
Lateral	8 (36%)	0 (0%)	3 (14%)	11 (50%)

Tibial Articular Cartilage				
Medial	22 (100%)	0 (0%)	0 (0%)	0 (0%)
Lateral	22 (100%)	0 (0%)	0 (0%)	0 (0%)

Femoral Articular Cartilage				
Medial	21 (95%)	1 (5%)	0 (0%)	0 (0%)
Lateral	22 (100%)	0 (0%)	0 (0%)	0 (0%)

**Table 2:** Inflammatory cytokines and chondrodegenerative markers evaluated on the day of ACL reconstruction [3, 4].

**Biomarker** **∗**	**Abbreviation**	**Volume ** **(Dilution)**	**CV**†	**LLOD**	**Number (**%**) below LLOD**^**‡**^
Cartilage oligomeric matrix protein (*μ*g/mL) Catalog #RD194080200, BioVendor, Asheville, NC	COMP	5 *μ*l (1:2500)	1.9%	0.4 ng/ml	0 (0%)

Synovial C-terminal crosslinked telopeptide of type II collagen (ng/mL) Cartilaps®, Catalog # AC-10F1, Immunodiagnostic Systems, Inc, Fountain Hills, AZ	CTX-II	40 *μ*l (None)	4.3%	0.20 *μ*g/L	0 (0%)

Urinary CTX-II normalized to creatinine concentration (ng/mmol) Cartilaps®, Catalog # AC-10F1, Immunodiagnostic Systems, Inc, Fountain Hills, AZ	uCTX-II	40 *μ*l (None)	3.3%	0.20 *μ*g/L	0 (0%)

Sulfated glycosaminoglycan (*μ*g/mL) Catalog # BP-004, Kamiya Biomedical Company, Seattle, WA	sGAG	25 *μ*l (None)	1.8%	N/A	0 (0%)

Interleukin-1 alpha (pg/mL) Catalog # K151RBD, Meso Scale Discovery, Rockville, MD	IL-1*α*	25 *μ*l (1:2)	9.9%	0.238 pg/ml	4 (18%)

Interleukin-1 beta (pg/mL) Catalog #K151QPD, Meso Scale Discovery, Rockville, MD	IL-1*β*	25 *μ*l (1:2)	5.7%	0.033 pg/ml	11 (50%)

Interleukin-1 receptor antagonist (pg/mL) Catalog # DRA00B, R&D Systems, Minneapolis, MN	IL-1ra	100 *μ*l (None)	4.4%	6.3 pg/ml	0 (0%)

Matrix metalloproteinase 3 plex (MMP-1, MMP-3, MMP-9) (pg/mL) Catalog # K15043C, Meso Scale Discovery, Rockville, MD	MMP-1	5 *μ*l (1:20)	3.5%	1.74 pg/ml	0 (0%)

	MMP-3	5 *μ*l (1:20)	4.5%	4.34 pg/ml	0 (0%)

	MMP-9	5 *μ*l (1:20)	3.5%	14.3 pg/ml	0 (0%)

N-terminal crosslinked telopeptide of type I collagen (nM BCE) Catalog # 9021, Osteomark ® NTx; Alere, Scarborough, ME	NTX-I	25 *μ*l (1:5)	2.3%	N/A	0 (0%)

Tumor necrosis factor-inducible gene 6 activity In-house ELISA assay developed by Kraus Laboratory[20]	TSG-6	4 *μ*l (1:100)	12.9%	1.0U	0 (0%)

*∗* All biomarkers measured in synovial fluid with the exception of urinary CTX-II.

† CV = mean interassay coefficient of variation.

‡ LLOD = lower limits of detection.

**Table 3:** Comparison of inflammatory cytokines and chondrodegenerative markers (mean ± standard deviation) evaluated on the day of ACL reconstruction between patients with KOOS QOL scores above and below the PASS threshold of 62.5 points.

**Biomarker**	**< PASS**	**≥ PASS**	**p** ^**a**^	**d** ^**e**^
N	6	16	-	-
Female/Male (n)	3/3	6/10	0.66	-
Steroid/Placebo (n)	4/2	12/4	> 0.99	-
Age (years)	18.0 ± 2.6	20.0 ± 4.5	0.42	-
BMI (kg/m^2^)	22.4 ± 2.9	24.8 ± 3.6	0.15	-
Graft (BTB/Hamstring)	5/1	13/3	> 0.99	-
Medial meniscus injury	5	9	0.35	-
Lateral meniscus injury	2	12	0.12	-
Bone bruise volume (mm^3^)	7.99 ± 8.93	11.07 ± 9.33	0.50	0.30
COMP (*μ*g/ml)	32.3 ± 12.5	39.3 ± 14.0	0.42	0.51
CTX-II (ng/ml)	1.57 ± 0.93	1.52 ± 1.97	0.38	0.03
uCTX-II^d^ (*μ*g/mmol)	5.72 ± 4.86	2.42 ± 2.09	0.08	0.99
sGAG (*μ*g/ml)	190.9 ± 69.9	264.7 ± 168.3	0.83	0.49
***IL-1*** **α** *** (pg/ml)***	***9.47 ± 7.65***	***2.21 ± 2.20***	***0.004***	***1.36***
IL-1*β*^c^ (pg/ml)	0.11 ± 0.13	0.45 ± 1.48	0.76	0.26
***IL-1ra (pg/ml)***	***2,593.2 ± 3,576.4***	***2,086.3 ± 5,507.0***	***0.03***	***0.10***
MMP-1 (ng/ml)	640.07 ± 81.58	394.06 ± 667.06	0.27	0.35
MMP-3 (ng/ml)	4,017.2 ± 4,576.41	2,532.80 ± 3,066.43	0.56	0.43
***MMP-9 (ng/ml)***	***30.99 ± 35.96***	***6.94 ± 10.30***	***0.01***	***1.07***
NTX-I (nM BCE)	30.3 ± 7.9	22.7 ± 7.1	0.055	0.97
TSG-6 (U)	286.4 ± 165.7	260.1 ± 157.3	0.83	0.11

^a^ Statistically significant differences denoted with ***bold and italics font.***

^b^ Number of patients in the corticosteroid or placebo group from the original randomized trial.

^c^ There was also no difference in the number of samples below LLOD between groups.

< Pass=3/6 versus ≥ PASS=8/16, p > 0.99.

^d^ u = urinary, the remaining biomarkers were measured in synovial fluid. Urinary CTX-II normalized to creatinine level (*μ*g/mmol).

^e^ Cohen's d effect sizes calculations were also performed in order to identify potentially clinically-meaningful findings within these pilot data, with d > 0.80 considered a large effect size.

**Table 4:** Comparison of inflammatory cytokines and chondrodegenerative markers (mean ± standard deviation) evaluated on the day of ACL reconstruction between patients with IKDC scores above and below the PASS threshold of 75.9 points.

**Biomarker**	**< PASS**	**≥ PASS**	**p** ^**a**^	**d** ^**e**^
N	9	13	-	-
Female/Male (n)	5/4	4/9	0.38	-
Steroid/Placebo^b^ (n)	6/3	10/3	0.66	-
Age (years)	18.9 ± 3.5	19.9 ± 4.6	0.82	-
BMI (kg/m^2^)	24.1 ± 3.9	24.2 ± 3.5	0.84	-
Graft (BTB/Ham)	8/1	10/3	0.62	-
Medial meniscus injury	7	7	0.38	-
Lateral meniscus injury	4	7	> 0.99	-
Bone bruise volume (mm^3^)	9.19 ± 7.94	10.94 ± 10.18	0.67	0.17
COMP (*μ*g/ml)	30.9 ± 10.2	41.9 ± 14.3	0.10	0.81
CTX-II (ng/ml)	1.16 ± 0.72	1.79 ± 2.16	0.92	0.37
uCTX-II^d^ (*μ*g/mmol)	3.92 ± 4.08	2.91 ± 2.77	0.62	0.30
sGAG (*μ*g/ml)	199.5 ± 70.1	275.8 ± 183.2	0.82	0.51
***IL-1*** **α** *** (pg/ml)***	***5.48 ± 4.56***	***3.29 ± 5.80***	***0.02***	***0.41***
IL-1*β*^c^ (pg/ml)	0.08 ± 0.09	0.55 ± 1.64	0.60	0.37
IL-1ra (pg/ml)	1,899.1 ± 3,014.8	2,621.5 ± 6,332.1	0.057	0.11
MMP-1 (ng/ml)	597.22 ± 689.63	366.96 ± 715.61	0.10	0.33
MMP-3 (ng/ml)	3,217.23 ± 4,074.67	2,744.09 ± 3,180.20	0.87	0.14
MMP-9 (ng/ml)	21.85 ± 31.59	7.71 ± 11.31	0.10	0.63
NTX-I (nM BCE)	26.6 ± 8.6	23.5 ± 7.5	0.33	0.40
TSG-6 (U)	260.9 ± 196.8	271.7 ± 129.3	0.53	0.40

^a^ Statistically significant differences denoted with ***bold and italics font.***

^b^ Number of patients in the corticosteroid or placebo group from the original randomized trial.

^c^ There was also no difference in the number of samples below LLOD between groups.

< Pass=5/9 ersus ≥ PASS=6/13, p > 0.99.

^d^ u = urinary, the remaining biomarkers were measured in synovial fluid. Urinary CTX-II normalized to creatinine level (*μ*g/mmol).

^e^ Cohen's d effect sizes calculations were also performed in order to identify potentially clinically-meaningful findings within these pilot data, with d > 0.80 considered a large effect size.

## Data Availability

The datasets used to support the findings of this study are restricted by the University of Kentucky Institutional Review Board in order to protect patient privacy. Data are available from the corresponding author for researchers who meet the criteria for access to confidential data.
